# Continuing the success of *Journal of Endocrinology* and *Journal of Molecular Endocrinology*: Editor-in-Chief handover

**DOI:** 10.1530/JOE-24-0305

**Published:** 2024-12-18

**Authors:** Martin Haluzik, Gabriela da Silva Xavier

**Affiliations:** ^1^ Diabetes Centre, Institute for Clinical and Experimental Medicine, and Institute of Medical Biochemistry and Laboratory Diagnostics, 1st Faculty of Medicine, Charles University, Prague, Czech Republic; ^2^ Department of Metabolism and Systems Science, University of Birmingham, Birmingham, UK

## Outgoing Editor-in-Chief

It has now been almost 12 years since I joined *Journal of Endocrinology* and *Journal of Molecular Endocrinology,* first as a Senior Editor and later, 5 years ago, as Co-Editor-in-Chief alongside Prof. Colin Farquharson and later with his successor Prof. Ruth Andrew. It has been a very interesting and exciting journey throughout the more and more rapidly changing landscape of scientific publishing and science in general. It was always a great pleasure and privilege being a part of these journals along with many outstanding and bright colleagues, and with great support from editorial colleagues at Bioscientifica and the Society for Endocrinology. In particular, I am very thankful to Publisher Millie Mark and Publishing Assistant Aidan Surgey, who were always able to keep me on time as much as possible with my duties in a very nice and very supportive way, despite my chronic tendency towards doing things at the very last, and mostly rather, late moment.

I enjoyed very much working with both Colin Farquharson and now with Ruth Andrew. Colin was always immensely helpful and supportive whenever I needed any help and input, especially in my early period as Co-Editor-in-Chief. Ruth, who joined us as Co-Editor-in-Chief a year ago, has brought a lot of inspirational and innovative ideas, along with great connections with many motivated and bright colleagues who have either published some great papers with us or joined our Editorial or Senior Editor boards. I would also like to thank very much Profs Barbara Clark and Morag Young who supported both journals as Deputy and Review Editors.

Together with Colin and later with Ruth, we faced some challenging times, such as the COVID-19 pandemic decreasing the number of submissions as a result of temporarily closed laboratories and other limitations during the COVID-19 pandemic. Another significant and ongoing challenge that we have had to deal with, and are still dealing with, is the increasing number of ‘paper mill’ submissions based on fabricated data, which challenge the long-term scientific integrity and reputation of our journals. To prevent that, we have created a system that makes sure that submissions of this nature are identified early and rejected at the level of Co-Editor-in-Chief preselection.

I am very glad that, together with other Co-Editors-in-Chief, we could introduce numerous changes and innovations to ensure an ongoing success of both of our journals. In addition to high-quality original papers and reviews, we have prepared several very successful anniversary or topic-focused issues, such as the 100-year anniversary of insulin discovery, or the issue focused on incretins’ physiology and therapeutic potential, to name a couple out of my area of expertise. We were also able to create a leadership and development award programme aiming to identify and develop the future leaders of the Society for Endocrinology, where two awardees of this programme could join the Senior Editorial Board as Early Career Editors. As a part of this role, our younger colleagues gained experience of peer reviewing (as an editor and as a reviewer), hot topic selection, promotional activities and marketing feedback, along with participation and involvement in social media campaigns. We have also commissioned a ‘Rising Stars’ collection for *Journal of Endocrinology* and *Journal of Molecular Endocrinology*. The involvement of young researchers along with commissioning high-quality articles from early- and mid-career researchers who are not yet fully established in their field remains an ongoing priority of our journals. We hope that these efforts will contribute to additional recognition for young researchers amongst their peers and help advance their careers.

Now, it is time to hand over my duties to Prof. Gabriela da Silva Xavier along with Prof. Ruth Andrew. I have no doubts that Gabriela is a perfect match for this role with her expertise in pancreatic islet biology, energy homeostasis and fuel-sensing mechanisms that may play a role in diabetes and obesity. Furthermore, she will bring along her previous experience from her work in other editorial boards in the past. I have absolutely no doubt that Prof. Gabriela da Silva Xavier will markedly contribute to maintaining and further improving the position of both *Journal of Endocrinology* and *Journal of Molecular Endocrinology* as leading global journals in endocrine and metabolic research, in both experimental and translational science. I wish both journals the best of luck, and I will be always happy to submit our papers or serve as a peer reviewer for these journals in the future.

Martin Haluzík

## Incoming Editor-in-Chief

It is a great privilege to be selected as the new Co-Editor-in-Chief of *Journal of Endocrinology* and *Journal of Molecular Endocrinology*, and I am very much looking forward to working with the authors, reviewers and colleagues at Bioscientifica and the Editorial Board for the journals. I would like to thank my predecessor, Prof. Martin Haluzík, for his enormous contribution to the publications, steering us safely through the increasingly challenging scientific publication landscape. I have huge shoes to fill and am keenly aware of my responsibilities to the community served by the two journals of the Society for Endocrinology. I hope to put the experience I gained from serving on the editorial boards of other journals to good use and am very much looking forward to learning from, and working with, Prof. Ruth Andrew, my fellow Co-Editor-in-Chief.

Our ability to probe biological systems is improving in leaps and bounds, and we are ever more able to address complex questions that may have been too challenging to properly address in the past. The exchange of ideas and knowledge from a wide field, a key function of scientific publication, is important to help us shape our research. Encouraging research that embraces the complexity conferred by diversity, forging consensus through multeity, can be challenging. However, there is much to be learnt from endeavours around the globe and we can achieve much more together. Bioscientifica, the publication arm of the Society for Endocrinology, has been doing a great job in being inclusive and promoting our work to researchers worldwide. For example, schemes such as Research4Life make publication more accessible for researchers from low- and middle-income countries, lowering the cost hurdle for the exchange of ideas and knowledge. There is still much to be done, and we hope to do more. As another step in this direction, I would like to champion the increased involvement of our more junior members of the research community, to encourage them to take a more active role in the work of the journal and to use their voice to help shape our community.

Please do get in touch to share your suggestions on how we can better serve our community.

Gabriela da Silva Xavier

